# More Than Color: Floral Color Polymorphism Is Associated With Scent Differentiation and Pollination Success in the Deceptive Orchid *Anacamptis pyramidalis*

**DOI:** 10.1002/ece3.74546

**Published:** 2026-11-02

**Authors:** Martina Sasso, Donata Cafasso, Vito Antonio Romano, Katharina Brandt, Manfred Ayasse, Salvatore Cozzolino

**Affiliations:** ^1^ Department of Biology University of Naples Federico II Naples Italy; ^2^ Department of Science University of Basilicata Potenza Italy; ^3^ Applied Zoology and Animal Ecology Freie Universität Berlin Berlin Germany; ^4^ Institute of Evolutionary Ecology and Conservation Genomics, Ulm University Ulm Germany

**Keywords:** floral phenotypes, food deception, genome size, plant–pollinator interactions, volatile organic compounds

## Abstract

Floral color polymorphism is widespread among flowering plants, yet the mechanisms underlying its maintenance remain poorly understood, particularly in food‐deceptive orchids where pollinator‐mediated selection may differ from that operating in rewarding species. Here, we investigated the relationship among floral color polymorphism, ploidy variation, floral scent composition, and pollination in the Mediterranean deceptive orchid *Anacamptis pyramidalis*. Cytotypes were determined by flow cytometry, and floral volatile organic compounds (VOCs) were analyzed using gas chromatography–mass spectrometry (GC–MS) in white‐ and pink‐flowered individuals from multiple populations. VOC profiles were compared using multivariate and univariate statistical approaches. Pollination success was assessed in mixed‐color diploid and tetraploid populations by quantifying the number of mature fruits produced per individual. Floral color morph was significantly associated with floral scent composition, accounting for approximately 6.5% of the total multivariate variation. Five VOCs differed significantly in relative abundance after correction for multiple comparisons, whereas total VOC emission did not differ significantly between morphs when cultivated in a common garden. No significant association between VOCs and cytotype was detected. White morphs consistently showed higher pollination success than pink morphs, producing significantly more fruits. By showing that floral color polymorphism is associated with both scent differentiation and differences in pollination success, our study highlights the importance of considering floral polymorphism as an integrated phenotype rather than a simple variation in flower pigmentation and helps explain its maintenance in the natural populations of this orchid.

## Graphical abstract

We estimated the interplay among floral color polymorphism, ploidy variation, floral scent composition, and fitness in natural polymorphic populations of a deceptive orchid. Our case study highlights the importance of considering floral polymorphism as an integrated phenotype and explains its maintenance in the natural populations of this orchid.

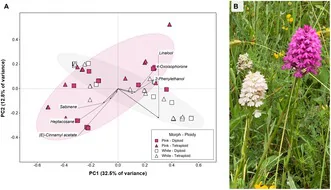

## 1 Introduction

Floral traits involved in pollinator attraction, such as flower color, shape, and scent, are often subject to strong pollinator‐mediated selection because pollinators use these floral signals during flower recognition and foraging (Stebbins 1970; Herrera et al. 2002; Schiestl and Johnson 2013). Pollinators frequently exhibit flower constancy and learning behavior that favor familiar floral phenotypes, potentially reducing the frequency of conspicuous variants within populations (Chittka et al. 1999; Waser 1986). Nevertheless, floral color polymorphism is widespread among flowering plants and has long attracted the attention of evolutionary ecologists because it raises the question of how alternative floral phenotypes are maintained within populations despite selection by pollinators (Arida et al. 2025; Warren and Mackenzie 2001; Clegg and Durbin 2000; Galen 1999). Despite decades of research, the mechanisms responsible for the maintenance of floral color polymorphism remain debated and are thought to involve multiple, non‐mutually exclusive processes (Sapir et al. 2021).

The maintenance of floral color polymorphism may be particularly intriguing in food‐deceptive orchids. Unlike rewarding species, deceptive orchids provide no nectar reward to pollinators, which rapidly learn to avoid unrewarding flowers after unsuccessful visits (Heinrich 1975; Dukas and Real 1993; Raine and Chittka 2007; Aragón and Ackerman 2004; Jersáková et al. 2006). Generally, deceptive orchids often exhibit elevated inter‐individual variation in floral traits (Sabat and Ackerman 1996; Salzmann et al. 2007). The traditional hypothesis proposes that the elevated phenotypic polymorphism of deceptive orchids reduces pollinator avoidance learning, thereby increasing pollinator attraction and reproductive success (Heinrich 1975; Nilsson 1992; Smithson and Macnair 1997; Ferdy et al. 1998). Under these conditions, rare floral variants may gain a selective advantage because they are less likely to be recognized and avoided by pollinators (Smithson and Macnair 1997; Ferdy et al. 1998). Consequently, negative frequency‐dependent selection has been proposed as a mechanism contributing to the maintenance of floral polymorphism in deceptive orchids (Gigord et al. 2001), although empirical evidence remains mixed among species (Juillet and Scopece 2010). In *Orchis mascula*, the presence of rare white‐flowered individuals has been shown to increase the pollination success of neighboring colored morphs, suggesting that rare morphs may alter pollinator responses to floral displays and contribute to the maintenance of floral polymorphism (Dormont et al. 2010; Schatz et al. 2013). In addition to visual signals, floral scent represents a major component of plant–pollinator communication (Raguso 2008). Floral scent consists of complex blends of volatile organic compounds (VOCs) belonging to multiple biosynthetic classes, including terpenoids, benzenoids, phenylpropanoids, and fatty acid derivatives (Knudsen et al. 2006; Raguso 2008; Dudareva et al. 2013). Pollinators can detect and discriminate among floral scent profiles and integrate olfactory and visual information during flower recognition and foraging decisions (Ayasse et al. 2011; Schiestl 2015).

As for flower color and shape, floral scent is often subject to strong pollinator‐mediated selection because pollinators rely on olfactory cues during flower recognition and foraging (Schiestl and Johnson 2013). Because floral color and volatile compounds are produced through interconnected secondary metabolic pathways (Dudareva et al. 2013), color morphs within species may differ not only visually but also chemically, exhibiting shifts in floral scent composition or in the relative abundance of specific VOCs (Majetic et al. 2007). Genetic factors may also influence floral scent composition and emission patterns. Polyploidization is known to alter plant metabolism through gene dosage effects, transcriptional regulation, and modifications of biosynthetic pathways, potentially leading to shifts in floral phenotype, including changes in floral scent composition and other floral traits compared with diploid individuals (Doyle et al. 2008; Chen 2010). Floral signaling and scent composition have been found to differ between cytotypes (Schlager et al. 2025; Gross and Schiestl 2015), potentially through gene‐dosage effects and altered regulation of VOC biosynthetic pathways (Picazo‐Aragonés et al. 2020).

The interactions between floral color and scent may also influence the learning‐mediated responses of pollinators. Experiments in controlled conditions with bumblebees have shown that olfactory cues can facilitate color discrimination and learning. Scent differentiation among color morphs could therefore influence the generalization of learned avoidance and potentially modify frequency‐dependent pollinator responses (Kunze and Gumbert 2001; Leonard et al. 2011). However, empirical studies that simultaneously investigate the interplay among floral color polymorphism, ploidy variation, and floral scent composition under natural conditions remain scarce.

*Anacamptis pyramidalis* (L.) Rich is a widespread Mediterranean food‐deceptive orchid (Delforge 2006) that represents an ideal study system for investigating the relationships among floral color polymorphism, ploidy variation, and floral scent (Pegoraro et al. 2016). This orchid produces a pyramidal inflorescence composed of small flowers bearing a broad labellum and a long, nectarless spur. It is pollinated primarily by diurnal Lepidoptera, particularly butterflies such as *Aporia crataegi*, *Maniola jurtina*, and *Melanargia galathea*, together with day‐flying moths of the genus *Zygaena* (e.g., *Z. filipendulae*, *Z. minos*, and *Z. carniolica*), which represent its main pollinators throughout its distribution (Nazarov and Efetov 1993; Lind et al. 2007; Claessens and Kleynen 2011). Unlike many orchid species in which white morphs occur only sporadically, the *A. pyramidalis* populations often contained relatively high frequencies of white‐flowered individuals, indicating that these morphs may not represent occasional developmental anomalies but rather be maintained within natural populations. The coexistence of contrasting floral color morphs suggests that color variation may be maintained if it confers some fitness advantage, such as increased pollinator visitation (Gigord et al. 2001; Jersáková et al. 2006). Such effects may arise directly from color differences or indirectly through associations between color and other floral traits involved in plant–pollinator interactions, particularly floral scent composition (Dormont et al. 2014). The *A. pyramidalis* populations studied here contained both white‐ and pink‐flowered individuals (Figure 1B) and comprised diploid and tetraploid cytotypes, providing an opportunity to investigate the relationships among floral color, ploidy level, scent composition, and pollination. Specifically, we asked: (i) whether floral color polymorphism and ploidy level influence overall floral scent composition; (ii) which volatile compounds and chemical classes underlie any scent differentiation associated with floral color; (iii) whether color morphs differ in total floral VOC emission; and (iv) whether floral color polymorphism is associated with differences in pollination success.

**FIGURE 1:**
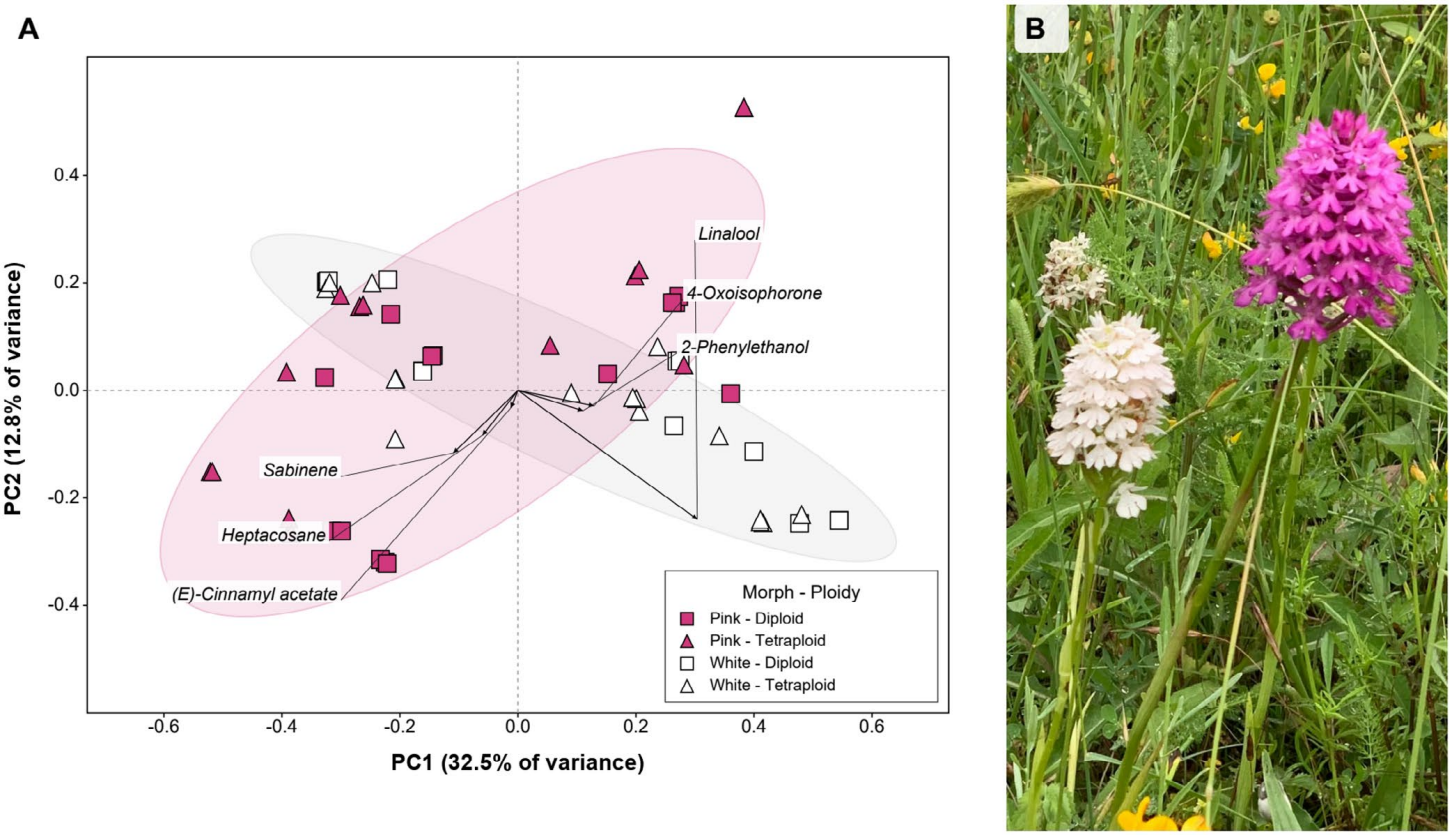
(A) Principal component analysis of Hellinger‐transformed floral VOC relative‐abundance data from white‐ and pink‐flowered morphs of *A. pyramidalis* in the two Basilicata populations. Colors indicate floral color morphs, and symbols indicate cytotypes. Shaded ellipses are descriptive visual guides and do not represent 95% confidence regions. Vectors represent the six compounds with the largest absolute loadings on PC1. Axis labels indicate the percentage of variance explained. (B) White‐ and pink‐flowered individuals at Vietri di Potenza.

## 2 Materials and Methods

### 2.1 Study Populations and Field Sampling

Floral scent of *A. pyramidalis* was sampled from two natural populations in Basilicata (Italy), each containing both white‐ and pink‐flowered individuals. The populations differed in cytotype, with Vietri di Potenza consisting of diploid plants (Table 1) and Diga del Camastra of tetraploid plants (V. A. Romano, pers. obs.). This design enabled comparisons between floral color morphs within each cytotype, although cytotype effects could not be fully separated from population identity. In addition, a diploid pink‐flowered population from Abruzzo and a tetraploid white‐flowered population from Tuscany were included as comparative reference populations (Table 1). To estimate the relative frequencies of the two color categories, white and pink flowering individuals were counted within an approximately 100 m radius of a central point in each of the two mixed‐color Basilicata populations. To minimize environmental heterogeneity and standardize sampling conditions, 95 individuals from the Vietri di Potenza population (Basilicata), representing both floral color morphs, were collected and cultivated in pots in common garden conditions, and their scent was analyzed during the following flowering season. After analysis, the plants were reallocated to the original spots.

**TABLE 1:** Geographic location, cytotype, floral color morph frequencies and number of analyzed individuals in *A. pyramidalis* populations investigated in the study.

Population	Region	Cytotype composition	Floral color	Relative frequency among counted extreme color phenotypes (%)	Number of analyzed individuals
Diga del Camastra	Basilicata	Tetraploid	Mixed	Pink 66.5% White 33.5%	31
Vietri di Potenza	Basilicata	Diploid	Mixed	Pink 55.9% White 44.1%	27
Palena	Abruzzo	Diploid	Monomorphic	Pink 100%	23
Greve in Chianti	Tuscany	Tetraploid	Monomorphic	White 100%	22

*Note:* For the mixed‐color populations, frequencies were calculated using only fully white and pink flowering individuals; rare, intermediate color phenotypes were excluded from the counts.

### 2.2 Cytotype Determination

All individuals included in the floral scent analyses were assigned to a cytotype by flow cytometric analysis of pollinia‐derived nuclei. Pollinia were used because they provide more reliable estimates of genome size and ploidy in orchids (Trávníček et al. 2015). In detail, fresh pollinia were collected from flowering individuals (soon after floral volatile collection) and processed following a standardized protocol (Kron et al. 2021). The ploidy level was determined by using the CyStain PI Absolute P kit (Sysmex Partec GmbH, Görlitz, Germany) for nuclear isolation and propidium iodide (PI) staining, according to the manufacturer's protocol. Flow cytometric analyses were performed on a Sysmex CyFlow Cube flow cytometer (Sysmex Partec GmbH, Görlitz, Germany).

### 2.3 Floral Volatile Collection and Chemical Analyses

Floral volatiles were collected using a dynamic headspace adsorption technique following Dötterl and Jürgens (2005). Individual inflorescences were enclosed in polytetrafluoroethylene (PTFE) bags and sampled for 4 h using ChromatoProbe quartz microvials (15 mm × 2 mm i.d.) filled with 1.5 mg Tenax‐TA (60–80 mesh) and 1.5 mg Carbotrap (20–40 mesh), fixed with glass wool. Purified air was supplied through activated charcoal filters (D.U.B.I. GmbH, Vienna, Austria), and airflow was maintained at 200 mL min^−1^ using a vacuum pump (PAS‐500, SPECTREX, Redwood City, CA, USA). Blank controls were collected under identical sampling conditions (same PTFE bags, adsorbent tubes, airflow, and sampling duration) but without plant material to verify the absence of background contamination. Field VOC sampling in the natural populations was conducted between 27 May and 4 June 2024 under partly sunny conditions, with temperatures ranging from 26°C to 28°C. Sampling started between 08:00 and 08:30 h. Equal numbers of white‐ and pink‐flowered individuals were sampled on each sampling day to balance color morphs across collection dates. Inflorescences were selected at a comparable flowering stage, with well‐open flowers and an approximately similar extent of floral display. Sampling was restricted to individuals with white or pink flowers; a few rare individuals with intermediate pale‐pink colouration were excluded. Before VOC collection, flowers were inspected to verify that their pollinia were still present, and flowers showing pollinia removal were excluded from sampling. Plants remained accessible to flower visitors before sampling; therefore, this screening did not exclude previous visits without pollinia removal or pollen deposition on the stigma.

Volatile compounds trapped on the adsorbent filters were analyzed at the Chemical Ecology lab (University of Ulm) using an Agilent 7890B gas chromatograph coupled to an Agilent 5977A mass‐selective detector (Agilent Technologies, Germany). The instrument was equipped with an HP‐5MS fused‐silica capillary column (5% diphenyl, 95% dimethylpolysiloxane; 30 m, inner diameter 0.25 mm, film thickness 0.25 μm). Samples were thermally desorbed using a GERSTEL Thermal Desorption system (TDU) and a cold‐injection apparatus (CIS 4C, Gerstel; initial temperature of −20°C; GERSTEL GmbH & Co. KG, Mülheim an der Ruhr, Germany) was used. The TDU temperature program was 40°C for 1 min, 100°C min^−1^ to 300°C, and a 1‐min hold at 300°C. Desorption was performed in splitless mode. The CIS temperature program was −20°C, followed by heating to 310°C at 12°C s^−1^ and a 4‐min hold at 310°C. Helium was used as the carrier gas at a constant column flow of 1.0 mL min^−1^. The GC oven temperature program was 40°C for 1 min, 6°C min^−1^ to 250°C, and a 30‐min hold at 250°C. The MSD transfer line, ion source, and quadrupole temperatures were maintained at 250°C, 230°C, and 150°C, respectively. Mass spectra were acquired in electron‐ionization mode at 70 eV over a mass range of m/z 35–450.

Chromatographic data were processed and compounds were identified using *GCMSsolution 4.12 SU2* (Shimadzu version 2.72, Shimadzu Corporation, Kyoto, Japan). Only well‐resolved chromatographic peaks exceeding a minimum peak‐height integration threshold of 300,000 were considered for subsequent analyses. Compound identities were assigned by comparing mass spectra and retention indices with commercial libraries (MassFinder 4, NIST11, Wiley Registry 9th Edition) (Adams 2007). A comparison of measured retention indices with literature values for selected compounds is provided in Table S2. Blank controls collected under identical sampling conditions were analyzed together with floral samples. Compounds identified as common analytical contaminants, including substances originating from adsorbent materials, sampling bags, laboratory equipment, or ambient air, as well as compounds consistently detected in blank samples, were excluded from subsequent analyses. In addition, authentic reference standards (Sigma‐Aldrich, Merck) were prepared from 1 mg mL^−1^ stock solutions, diluted to 20 μg mL^−1^ working solutions, and analyzed under the same chromatographic conditions as floral samples to confirm compound identity. Standards included representative alcohols, aldehydes, ketones, aromatic compounds, monoterpenes, sesquiterpenes, and *n*‐alkanes (see Table S1 for the complete list). For all samples, chromatographic peak areas were integrated to obtain the total ion current (TIC) for each detected compound. Relative VOC abundances (%) were calculated from TIC peak areas for all samples.

For the 95 potted plants analyzed during the following flowering season, absolute VOC emission rates were quantified using external calibration curves generated for the most abundant floral VOCs identified in the previous analyses. Calibration curves were constructed from three concentrations of authentic reference standards analyzed under the same chromatographic conditions as floral samples, and VOC emission rates were expressed as ng h^−1^. Total floral scent emission was calculated as the sum of the quantified amounts of all detected VOCs over the standardized 4‐h sampling period. For all remaining populations, analyses were based exclusively on relative VOC abundances.

### 2.4 Plant Pollination Success

*A. pyramidalis* does not undergo autonomous self‐pollination and requires insect‐mediated pollen transfer (Tremblay et al. 2005). Interestingly, Darwin (1862) was the first to describe a delayed bending movement of the *A. pyramidalis* pollinia following their removal by a pollinator that avoids self‐pollination. Fruit set was assessed in both diploid and tetraploid Basilicata populations (i.e., Vietri di Potenza and Diga del Camastra).

A total of 245 individuals belonging to the two floral color morphs were marked and monitored throughout the reproductive season. Pollination success was assessed following the protocol described by D'Aria et al. (2024), quantified as the number of mature fruits produced per individual at the end of the reproductive season (i.e., late June). The total number of flowers produced at anthesis was also recorded for each plant.

### 2.5 Statistical Analyses

Statistical analyses were performed using relative abundance (%) data obtained from GC–MS analyses of floral VOCs. Samples from the complete sampling campaign were analyzed as a single pooled dataset. VOC profiles were converted to proportions summing to one within each sample and Hellinger‐transformed by taking the square root of each proportion. This transformation reduced the influence of dominant compounds and made Euclidean ordination of the VOC profiles appropriate.

Patterns of variation in floral scent composition were explored using principal component analysis (PCA) performed on centered, unscaled Hellinger‐transformed data. PCA scores were used to visualize compositional relationships among individuals and floral color morphs. Compounds associated with the principal axes were identified descriptively from their PCA loadings.

Differences in overall VOC composition were evaluated using permutational multivariate analysis of variance (PERMANOVA), based on Bray–Curtis dissimilarities calculated from untransformed relative‐abundance data. The model included floral color morph, cytotype group, and their interaction. Model terms were tested sequentially, with floral color morph entered first, using 9999 permutations. Pseudo‐*F* statistics, *R* ^2^ values, and permutation‐based *p*‐values were calculated for each term.

To assess differences in within‐morph variability, we tested the homogeneity of multivariate dispersion using distances to group spatial medians in principal‐coordinate space derived from Bray–Curtis dissimilarities. Distances were adjusted for sample size, and significance was assessed using 9999 permutations of model residuals. We also fitted an additive PERMANOVA model with population entered before color morph, restricting permutations within populations to test the color association after accounting for population. Population and cytotype were not included as separate predictors because they coincided in the two study populations. Finally, sensitivity to individual samples was assessed by repeating the original color morph × cytotype model after omitting each individual in turn, using 999 permutations for each leave‐one‐out analysis. A random seed of 42 was used for these analyses.

A descriptive similarity percentage (SIMPER) analysis was performed on untransformed relative‐abundance data to quantify the contribution of each VOC to the average Bray–Curtis dissimilarity between white and pink morphs. The analysis included all 105 VOC variables from the two Basilicata populations. Contributions were expressed as percentages of the average between‐morph dissimilarity. These SIMPER contributions describe differences between individual scent profiles and should not be interpreted as a breakdown of variation associated with color morph in the PERMANOVA, that is, a compound may contribute strongly to this dissimilarity because it varies among individuals, including those of the same color morph.

For compound‐level analyses, relative abundances of all 105 detected VOCs were compared between white‐ and pink‐flowered morphs using two‐sided Mann–Whitney *U* tests. To control the false discovery rate across these simultaneous comparisons, *p*‐values were adjusted using the Benjamini–Hochberg procedure. Adjusted values are reported as FDR *q*‐values.

Selected VOCs were also compared between diploid and tetraploid sample groups using two‐sided Mann–Whitney *U* tests. These comparisons were treated as exploratory, and their unadjusted *p*‐values were reported.

To investigate broader patterns of scent differentiation, the 105 VOC features were assigned to mutually exclusive categories primarily according to their functional groups. Terpene alcohols were included among alcohols, terpene hydrocarbons among hydrocarbons, and lactones among esters. Indole was assigned to the N‐containing heterocycles category. Features identified only as an alcohol or an aldehyde were retained in their respective functional categories. Features whose classification remained unresolved, including those labeled Phenyl alcohol, Precoc‐1‐ene and Benzoate, were retained in the unclassified category. For each individual, relative abundances were summed within each category, retaining all 105 features in the relative‐abundance denominator. The eight categories were compared between white‐ and pink‐flowered morphs using two‐sided Mann–Whitney *U* tests. *p*‐values were adjusted using the Benjamini–Hochberg procedure across these eight comparisons and are reported as *q*‐values.

Total VOC emission was calculated for each individual by summing the absolute emission rates of the measured compounds and was compared between floral color morphs using a two‐sided Mann–Whitney *U* test.

Heatmaps were constructed from mean relative abundances of selected VOCs calculated for each population–morph group. Values were standardized separately for each compound as *z*‐scores across the four groups. Similarity among group‐level mean VOC profiles was summarized descriptively using Pearson correlation coefficients; these correlations were used for exploratory visualization rather than formal hypothesis testing.

Pollination success, quantified as the number of fruits produced per individual, was compared between white‐ and pink‐flowered morphs using two‐sided Mann–Whitney *U* tests, both overall and within each mixed‐color population. Fruit production was also compared between populations using two‐sided Mann–Whitney *U* tests. Total flower number was compared between groups using Welch's two‐sample *t*‐tests.

Analyses were implemented in Python 3.12 using custom scripts based primarily on pandas and NumPy.

## 3 Results

### 3.1 Cytotype Determination and Color Morph Frequency in the Populations

The cytotype assignment of all individuals selected for floral scent analyses was confirmed by flow‐cytometric analysis. In detail, all plants from Vietri di Potenza and Palena populations were diploid, whereas all plants from Diga del Camastra and Greve in Chianti populations were tetraploid. According to the visual census, white morphs accounted 44.1% at Vietri di Potenza and 33.5% at Diga del Camastra, whereas pink morphs represented 55.9% and 66.5%, respectively (Table 1).

### 3.2 Composition of Floral Scent Blends

In the headspace samples of *A. pyramidalis* , 105 VOC features were detected. The functional classification comprised 21 alcohols, 10 aldehydes, 10 carboxylic acids, 19 esters including lactones, 21 hydrocarbons, eight ketones, and one N‐containing heterocycle (Indole); 15 features remained unclassified. These counts include features assigned to a functional category without a resolved compound identity. Alcohols and hydrocarbons were the most represented categories in terms of the number of features, whereas carboxylic acids accounted for the highest mean summed relative abundance in both color morphs (Figure 3). No compounds were detected exclusively in either white‐ or pink‐flowered individuals, although several VOCs occurred only sporadically and in trace amounts. Consequently, differences between floral color morphs reflected quantitative variation in the relative abundance of shared compounds rather than qualitative differences in compound occurrence. Measured and literature retention indices for selected VOCs are reported in Table S2.

At the category level, alcohols had a higher summed relative abundance in white than in pink morphs (18.16% ± 10.37% vs. 10.70% ± 8.88%; mean ± SD; *q* = 0.034), whereas hydrocarbons had a higher summed relative abundance in pink than in white morphs (15.17% ± 7.59% vs. 10.28% ± 2.93%; *q* = 0.024). None of the other categories differed significantly between morphs after correction for multiple comparisons (all *q* > 0.05; Figure 3).

Although white and pink morphs shared the same overall VOC repertoire, their relative‐abundance profiles showed partial differentiation associated with floral color. PCA performed on Hellinger‐transformed relative‐abundance data revealed substantial overlap between morphs along the first two axes (PC1 = 32.5%, PC2 = 12.8% of explained variance; Figure 1A). White morphs had a higher mean score on PC1 than pink morphs, but the two morphs did not form clearly separated groups.

The patterns observed in the PCA were further supported by PERMANOVA. PERMANOVA based on Bray–Curtis dissimilarities detected a statistically significant association between floral color morph and VOC composition, although color explained only 6.5% of the total multivariate variation (pseudo‐*F*_1,54_ = 3.84, *R* ^2^ = 0.065, *p* = 0.0089; 9999 permutations). Neither the cytotype term (pseudo‐*F*_1,54_ = 0.99, *R* ^2^ = 0.017, *p* = 0.3679) nor the color morph × cytotype interaction (pseudo‐*F*_1,54_ = 0.64, *R* ^2^ = 0.011, *p* = 0.6597) was statistically significant.

Additional analyses assessed the robustness of the association between color morph and scent composition. A permutation test of multivariate dispersion did not detect a difference between morphs (PERMDISP, *F*_1,56_ = 0.022, *p* = 0.8894). In an additive PERMANOVA model accounting for population, the association with color remained statistically significant when permutations were restricted within populations (pseudo‐*F*_1,55_ = 3.88, *R* ^2^ = 0.065, *p* = 0.0075). Repeating the original model after excluding each individual in turn yielded color‐associated *R* ^2^ values of 0.057–0.073 and *p*‐values of 0.004–0.017. Thus, statistical significance was not dependent on any single individual.

Supplementary analyses of the Basilicata populations showed extensive overlap between diploid and tetraploid individuals in the PCA, and none of the selected VOCs differed significantly between cytotypes in the exploratory univariate comparisons (Figure S1). These comparisons do not separate cytotype effects from population effects.

Although floral color explained a small proportion of the total variation in VOC composition, it was the only factor showing a significant effect in the PERMANOVA.

### 3.3 Relevant VOCs Associated With Morph Differentiation

Linalool had a higher mean relative abundance in white morphs (*q* = 0.011), whereas Nonadecanol, (E)‐Cinnamyl acetate, Heptacosane and 6‐Methyl‐5‐hepten‐2‐one had higher mean relative abundances in pink morphs (*q* = 0.005, 0.007, 0.008, and 0.011, respectively). The descriptive SIMPER analysis of the relative‐abundance data identified Hexadecanoic acid (11.44%), Linalool (8.84%), Pentanal (5.84%), Nonanal (5.72%), Oleic acid (4.89%) and Octadecanoic acid (4.81%) as the largest contributors to the average Bray–Curtis dissimilarity between white‐ and pink‐flowered individuals across the two Basilicata populations. Together, these compounds accounted for 41.54% of the average between‐morph dissimilarity.

An exploratory comparison of morph‐associated VOCs in the two geographically distant reference populations (Tuscany and Abruzzo) revealed a similar qualitative pattern of scent differentiation (Figure S2). Because these populations were monomorphic and differed in both geographic origin and cytotype composition, this analysis was intended only to assess the consistency of color‐associated VOC signatures and not to infer population‐level or geographic effects. In particular, white morphs from Basilicata and Tuscany were both characterized by relatively high mean abundances of Linalool, Pentanal, and 4‐Oxoisophorone, whereas pink morphs from Basilicata and Abruzzo showed higher mean abundances of Heptacosane, Sabinene, and (E)‐Cinnamyl acetate. Taken together, these descriptive patterns suggest that several VOCs associated with floral color differentiation show broadly consistent abundance trends across geographically separated populations.

### 3.4 Comparison of Total VOC Emission

In plants cultivated in the common garden, white morphs showed a higher mean total VOC emission than pink morphs (357.5 ± 372.7 ng h^−1^, *n* = 48, and 240.0 ± 216.9 ng h^−1^, *n* = 47, respectively). However, total VOC emission did not differ significantly between floral color morphs (Mann–Whitney *U* = 1333.0, *p* = 0.128), suggesting that morph‐related scent differences involved VOC composition and the emission of specific compounds rather than overall VOC emission (Figure 4). Notably, two of the main floral VOCs showing morph‐related differences in relative abundance in the natural population of Vietri di Potenza, Linalool and 4‐Oxoisophorone, were also emitted at significantly higher rates by white than by pink morphs in the common garden analysis (28 plants per morph; two‐sided Mann–Whitney *U* tests: *U* = 584.5, Holm‐adjusted *p* = 0.0015 for linalool; *U* = 667.0, Holm‐adjusted *p* < 0.0001 for 4‐oxoisophorone).

### 3.5 Plant Pollination Success

Pollination success differed significantly between floral color morphs in both Basilicata populations. White morphs produced significantly more fruits than pink morphs overall (12.03 ± 7.33 vs. 7.07 ± 5.63 fruits per individual, respectively; Mann–Whitney *U* test, *p* = 4.62 × 10^−9^) (Figure 5). This pattern was observed in both Basilicata populations. At Vietri di Potenza, white morphs produced an average of 11.45 ± 7.50 fruits per individual (*n* = 83), whereas pink morphs produced 7.19 ± 5.90 fruits (*n* = 114; Mann–Whitney *U* test, *p* = 6.33 × 10^−6^). Similarly, at Diga del Camastra, white morphs produced 14.04 ± 6.49 fruits per individual (*n* = 24), compared with 6.50 ± 4.22 fruits in pink morphs (*n* = 24; Mann–Whitney *U* test, *p* = 4.38 × 10^−5^).

Overall, fruit production did not differ significantly between the two populations. Plants from Vietri di Potenza produced 8.98 ± 6.93 fruits (*n* = 197; Mann–Whitney *U* test, *p* = 0.131), whereas those from Diga del Camastra produced an average of 10.27 ± 6.62 fruits per individual (*n* = 48).

At Vietri di Potenza, white‐flowered individuals produced significantly fewer flowers than pink‐flowered individuals (36.80 ± 21.85 vs. 52.10 ± 25.28 flowers per plant, respectively; Welch's *t*‐test: *t* = −4.54, df = 189.27, *p* = 1.00 × 10^−5^). At Diga del Camastra, flower production did not differ significantly between white‐ and pink‐flowered individuals (57.96 ± 16.63 vs. 51.96 ± 15.91 flowers per plant, respectively; Welch's *t*‐test: *t* = 1.28, df = 45.91, *p* = 0.208). Therefore, the higher fruit production observed in white morphs cannot be attributed to a greater number of flowers.

## 4 Discussion

### 4.1 Floral Color Polymorphism and Variation in Floral Scent Composition

Floral color morph was significantly associated with floral scent composition, but accounted for only approximately 6.5% of the total multivariate variation. This small effect size, together with the substantial overlap in the PCA, indicates partial differentiation rather than clearly distinct scent compositions. No significant difference in multivariate dispersion was detected between morphs, and the association between color morph and scent composition remained significant when individual samples were omitted one at a time. These results suggest that the association was not dependent on any single individual, although they do not exclude the joint influence of multiple samples. The color effect also remained significant after accounting for population identity. Nevertheless, because cytotype and population identity were confounded in the two Basilicata populations, their effects cannot be separated. Further, even if sampling conditions were standardized, residual variation associated with flower age and visitation history may have contributed to variation among individual scent profiles.

Within these limitations, white and pink morphs differed significantly in overall floral scent composition, whereas diploid and tetraploid individuals showed extensive overlap and no consistent differences in either multivariate VOC profiles or the relative abundance of the principal morph‐associated compounds. More generally, our results indicate that differences between floral color morphs extend beyond floral pigmentation and are associated with coordinated differences in floral scent composition.

The limited influence of cytotype on scent is noteworthy because polyploidization can induce phenotypic changes with important ecological consequences, including altered interactions with pollinators (Segraves and Anneberg 2016). In some terrestrial orchids, particularly in *Gymnadenia*, cytotype divergence has been associated with differences in floral scent, pollinator assemblages, and reproductive isolation (Huber et al. 2005; Chapurlat et al. 2015; Gross and Schiestl 2015).

### 4.2 Morph‐Specific Scent Differentiation Involves Relevant VOCs Rather Than Differences in Total Scent Emission

Linalool had a significantly higher relative abundance in white morphs after correction for multiple comparisons. Although white morphs also showed higher mean relative abundances of 2‐Phenylethanol and 4‐Oxoisophorone, these differences were not statistically significant after correction (Table 2; Figure 2). Linalool and 2‐Phenylethanol are among the most widespread floral volatiles in angiosperms and are known to contribute to pollinator attraction, floral recognition and associative learning (Knudsen et al. 2006; Raguso 2008; Dudareva et al. 2013), whereas 4‐Oxoisophorone has been reported from the floral scent of several insect‐pollinated species and shown to elicit physiological and behavioral responses in flower‐visiting insects (El‐Sayed et al. 2018). This observation is of particular interest because *A. pyramidalis* is pollinated primarily by butterflies and day‐flying moths, with zygaenid moths representing its most important pollen vectors (Lind et al. 2007). These insects rely on both visual and olfactory cues during flower location and foraging (Raguso 2004, 2008; Stöckl and Kelber 2019). Although the behavioral responses of these pollinators to the VOCs identified here remain unknown, the higher abundance of some biologically relevant compounds in white morphs raises the possibility that differences in floral scent contribute to pollinator attraction or discrimination in combination with floral color.

**TABLE 2:** Relative abundances of selected floral volatile organic compounds in white‐ and pink‐flowered *A. pyramidalis* individuals from the two Basilicata populations.

Compound	RI	White morph (%), mean ± SD	Pink morph (%), mean ± SD	*q*
Linalool	1097.4	10.76 ± 8.87	3.36 ± 4.76	0.011
2‐Phenylethanol	1111.4	1.35 ± 3.14	0.48 ± 1.30	0.402
4‐Oxoisophorone	1139.6	2.51 ± 1.72	1.38 ± 1.23	0.137
Pentanal	705.0	7.25 ± 8.00	2.69 ± 2.15	0.483
(E)‐Cinnamaldehyde	1267.3	0.36 ± 0.56	0.24 ± 0.49	0.444
Nonadecanol	2273.2	0.33 ± 0.34	0.90 ± 0.56	0.005
(E)‐Cinnamyl acetate	1377.3	0.52 ± 0.56	1.57 ± 1.35	0.007
Heptacosane	2488.7	0.33 ± 0.34	1.17 ± 1.41	0.008
Sabinene	959.0	0.36 ± 1.20	1.80 ± 2.30	0.147
6‐Methyl‐5‐hepten‐2‐one	973.3	0.005 ± 0.014	1.81 ± 2.27	0.011
Hexadecane	1591.2	0.77 ± 0.79	1.32 ± 3.44	0.402
1‐Nonadecene	1685.9	1.00 ± 0.77	0.61 ± 0.56	0.483
Methyl nicotinate	1132.1	1.35 ± 1.08	1.06 ± 1.09	0.483
Decanoic acid	1346.8	0.002 ± 0.003	0.06 ± 0.22	0.106
Pentadecane	1500.2	0.09 ± 0.23	0.18 ± 0.26	0.483

*Note:* Values are mean relative abundance (%) ± sample standard deviation (SD), based on 29 individuals per morph. Comparisons were performed using two‐sided Mann–Whitney *U* tests with a tie‐corrected normal approximation and continuity correction. *p*‐values were adjusted across all 105 VOC variables using the Benjamini–Hochberg procedure. FDR‐adjusted *p*‐value (*q*‐values) indicate statistical significance at *q* < 0.05.

Abbreviation: RI, retention index.

**FIGURE 2:**
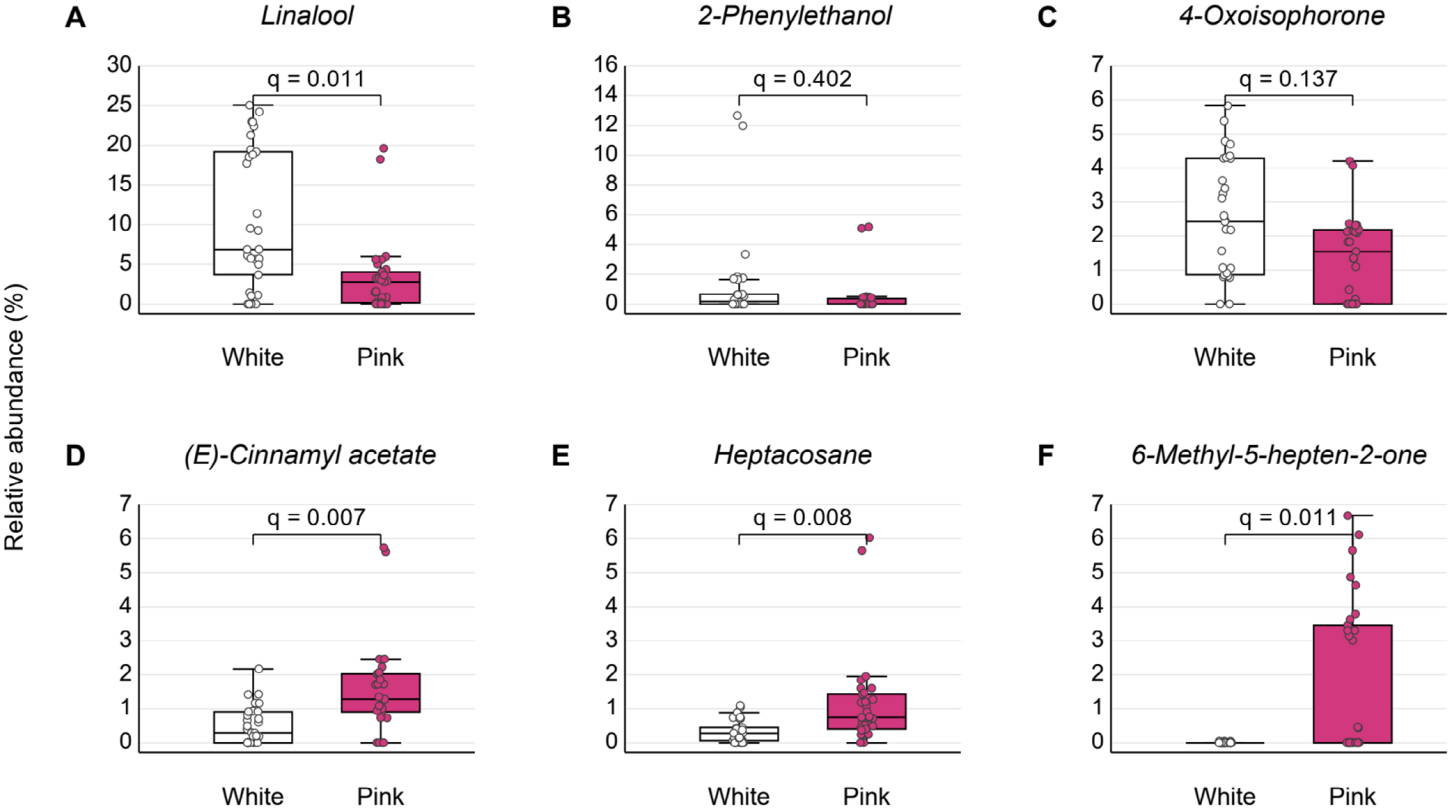
Relative abundances (%) of selected floral volatile organic compounds (VOCs) in white‐ and pink‐flowered morphs of *A. pyramidalis* from Basilicata (*n* = 29 per morph). Boxes show the interquartile range (25th–75th percentiles), horizontal lines indicate medians, whiskers extend to the most extreme values within 1.5 times the interquartile range, and points represent individual samples. Displayed *q*‐values are *p*‐values from two‐sided Mann–Whitney *U* tests adjusted using the Benjamini–Hochberg procedure across all 105 VOC variables. Linalool, (E)‐Cinnamyl acetate, Heptacosane and 6‐Methyl‐5‐hepten‐2‐one differed significantly between morphs after correction, whereas 2‐Phenylethanol and 4‐Oxoisophorone did not.

Differences between color morphs were not limited to individual VOCs but also involved broader variation in bouquet composition. White morphs showed a higher summed relative abundance of alcohols, whereas pink morphs showed a higher summed relative abundance of hydrocarbons, including terpene hydrocarbons (Figure 3). These differences remained statistically significant after correction for multiple comparisons. In contrast, total VOC emission did not differ significantly between morphs in the common garden comparison (Figure 4). Together, these findings indicate color‐associated differences in relative VOC composition without evidence of a corresponding difference in total emission under the common garden conditions examined.

**FIGURE 3:**
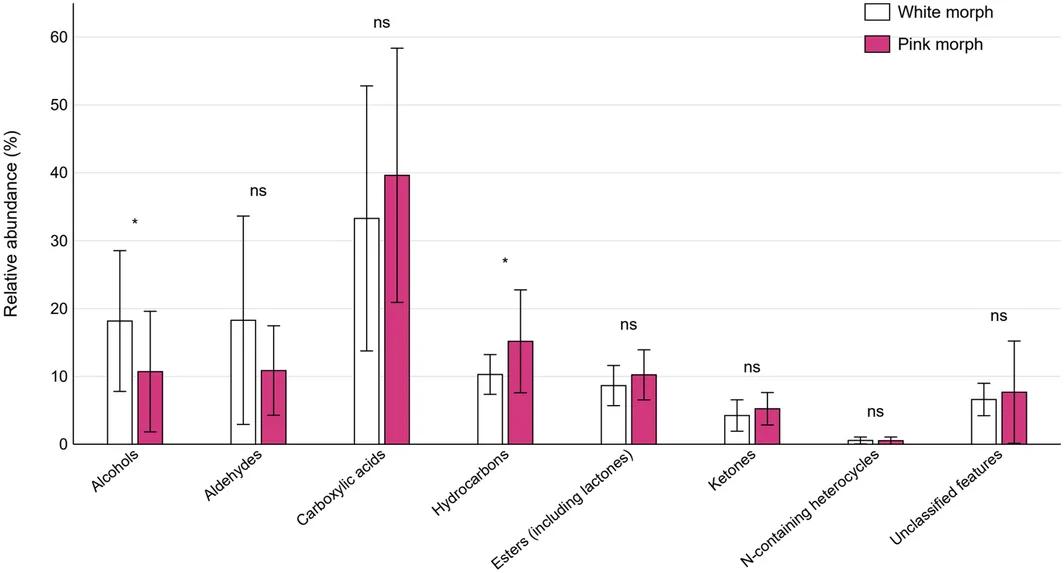
Relative abundances of VOC categories in white‐ and pink‐flowered morphs of *A. pyramidalis* from the two Basilicata populations (*n* = 29 individuals per morph). Bars represent mean summed relative abundances (%) and error bars indicate standard deviations. Each of the 105 VOC features was assigned to one category using a functional‐group classification; terpene hydrocarbons were included among hydrocarbons, terpene alcohols among alcohols, and lactones among esters. The N‐containing heterocycles category comprises Indole. Differences between morphs were assessed using two‐sided Mann–Whitney *U* tests, with Benjamini–Hochberg correction across the eight categories. Symbols refer to adjusted *p*‐values (*q*): *q ≤ 0.05; ns, *q* > 0.05. Alcohols were more abundant in white morphs (*q* = 0.034), whereas hydrocarbons were more abundant in pink morphs (*q* = 0.024).

**FIGURE 4:**
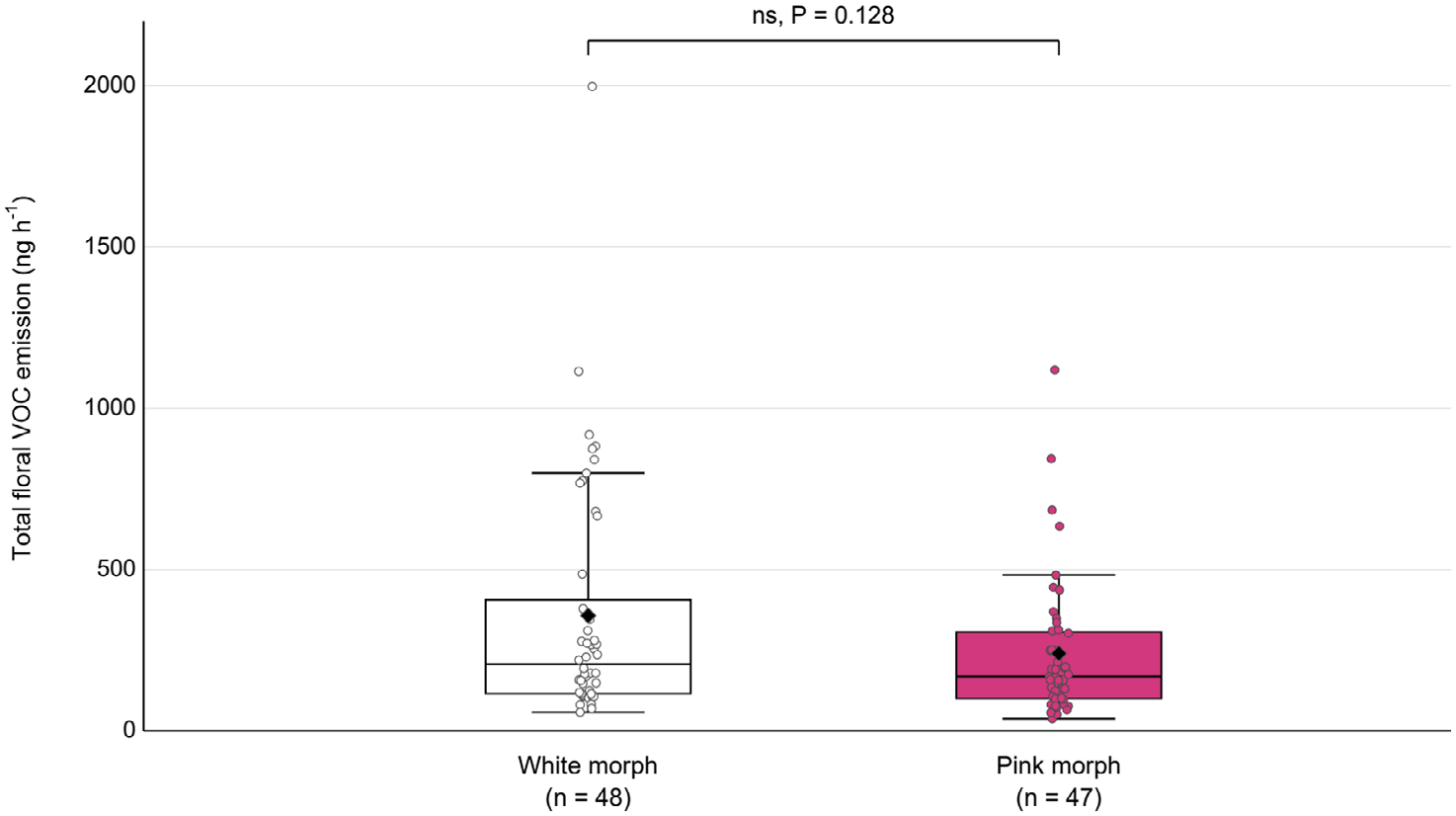
Comparison of the total floral VOC emission (ng h^−1^) in white and pink morphs of *A. pyramidalis* from potted plants of the Vietri di Potenza population. Boxes indicate the interquartile range, horizontal lines the median, whiskers 1.5 × interquartile range, and points individual plants.

Similar associations between floral color polymorphism and floral scent differentiation have been reported in both orchids and other flowering plants (Majetic et al. 2007; Dormont et al. 2014). Particularly relevant is the food‐deceptive orchid *Dactylorhiza romana*, in which color morphs differed in the relative abundance of Linalool and Benzaldehyde despite sharing the same major floral volatiles (Salzmann and Schiestl 2007). Likewise, white and pink morphs of *A. pyramidalis* shared most VOCs but differed consistently in the relative abundance of several biologically relevant compounds. Although the behavioral significance of these differences remains to be demonstrated, the similarity between these systems suggests that floral color polymorphism in deceptive orchids may frequently be associated with variation in both visual and olfactory signals rather than flower color alone.

The SIMPER results indicate that variation in the relative proportions of several shared compounds contributes to the observed scent differences between morphs. Hexadecanoic acid, Linalool, Pentanal, Nonanal, Oleic acid, and Octadecanoic acid together accounted for 41.54% of the average between‐morph dissimilarity.

Among these compounds, Linalool combined a substantial SIMPER contribution with a significantly higher relative abundance in white morphs (assessed separately using Mann–Whitney *U* tests with Benjamini–Hochberg correction), making it a candidate for future behavioral tests of pollinator discrimination. The exploratory comparison with Tuscany and Abruzzo revealed recurring abundance patterns for several selected VOCs across geographically separated populations (Figure S2). White‐flowered groups showed higher mean relative abundances of Linalool, Pentanal, and 4‐Oxoisophorone, whereas pink‐flowered groups showed higher mean relative abundances of Heptacosane and Sabinene. These similarities provide preliminary support for the recurrence of color‐associated scent patterns beyond the two mixed‐color Basilicata populations. However, additional polymorphic populations would be needed to distinguish color associations from population and cytotype effects.

### 4.3 Pollination Success and the Maintenance of Floral Color Polymorphism

White morphs consistently exhibited higher pollination success than pink morphs in both diploid and tetraploid populations, indicating that floral color polymorphism is associated with differences in plant reproductive success (Figure 5). Because floral scent composition and pollination success were measured in different individuals, our study cannot determine whether individual variation in floral scent directly predicts pollination success.

**FIGURE 5:**
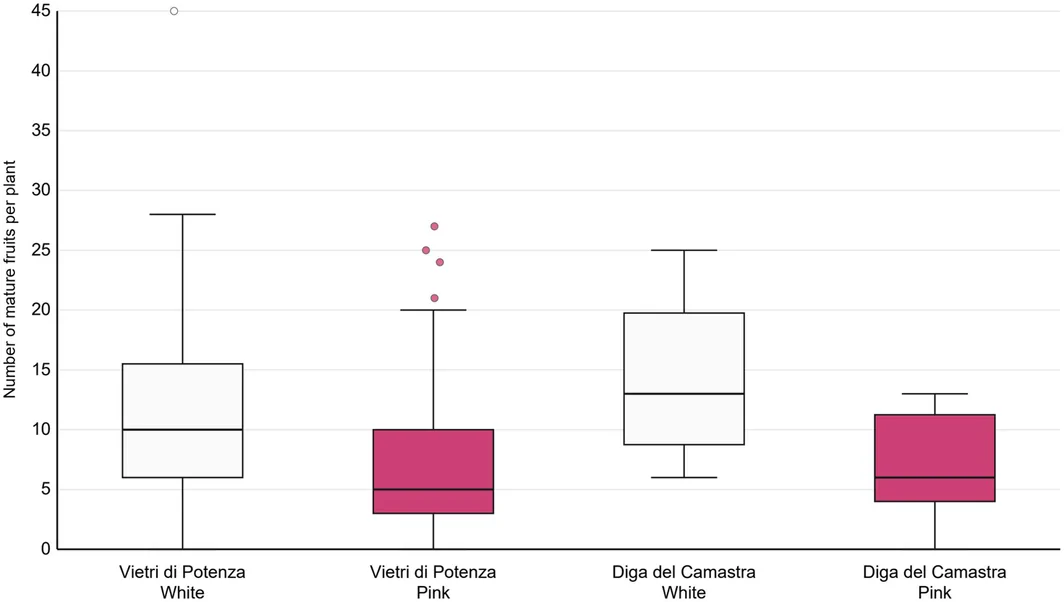
Pollination success of floral color morphs in the two Basilicata populations. Boxplots show the number of fruits produced per individual plant by white‐ and pink‐flowered morphs at Vietri di Potenza and Diga del Camastra. Points represent individual plants, horizontal lines indicate medians, boxes represent interquartile ranges, and whiskers 1.5 × interquartile range.

In food‐deceptive orchids, successful pollination depends on attracting naïve pollinators while minimizing learned avoidance following unrewarding visits (Jersáková et al. 2009). The higher fruit production of white morphs should be considered in relation to their frequencies within the studied populations. White‐flowered individuals were less frequent than pink‐flowered individuals at both Vietri di Potenza and Diga del Camastra, although they were not rare in either population (Table 1). Under the negative frequency‐dependent selection hypothesis, pollinators may have fewer opportunities to learn to avoid less frequently encountered floral phenotypes. The higher fruit production of white morphs is therefore compatible with a frequency‐dependent advantage. However, our observational comparison does not establish whether reproductive success decreases with increasing morph frequency and thus cannot demonstrate negative frequency‐dependent selection.

Floral scent differentiation could further influence the learning processes underlying such frequency‐dependent responses. Kunze and Gumbert (2001) showed that olfactory cues can facilitate discrimination between rewarding and unrewarding artificial flowers in bumblebees, and that the presence of scent can enhance color discrimination even when scent itself does not distinguish the flower types. Similarly, Leonard et al. (2011) found that scent facilitated color learning and reduced uncertainty in color discrimination. These findings suggest that the scent differences associated with color morphs in *A. pyramidalis* could influence how pollinators learn and generalize avoidance following unrewarding visits. If perceptible to pollinators, these scent differences might reduce the transfer of learned avoidance from the more frequent pink morph to the less frequent white morph.

Conversely, enhanced discrimination could also facilitate recognition and avoidance of both morphs as pollinators gain experience. These possibilities remain untested, also because the cited experiments involved bumblebees, whereas *A. pyramidalis* is pollinated predominantly by Lepidoptera. Finally, white flowers may also remain more detectable under low‐light conditions, potentially increasing visitation by crepuscular or nocturnal pollinators (White et al. 1994; Kelber 2006).

Whether enhanced visual detectability, differences in floral scent composition, or the interaction between these signals contributes to the higher reproductive success of white morphs remains unknown and requires experimental investigation.

## 5 Conclusion

Our study provides evidence that floral color polymorphism in *A. pyramidalis* is associated with coordinated variation in multiple floral traits, including floral scent composition and pollination success, extending beyond differences in flower pigmentation alone. More broadly, our results highlight the importance of considering multimodal floral signaling when investigating the maintenance and evolution of floral polymorphism, particularly in deceptive orchids, where interactions between visual and olfactory signals may influence pollinator behavior. Although the mechanisms underlying the higher pollination success of white morphs remain unresolved, the consistent association between floral color, floral scent composition and pollination success highlights the need for future studies combining long‐term demographic monitoring, measurements of lifetime reproductive success, experimental manipulation of color morph frequencies within populations, and detailed behavioral observations of pollinators. Such studies will be essential to determine whether floral color polymorphism in *A. pyramidalis* is maintained by negative frequency‐dependent selection, pollinator‐mediated directional selection, or other selective processes.

## Author Contributions

**Martina Sasso:** data curation (equal), formal analysis (equal), investigation (equal), methodology (equal), software (equal), validation (equal), writing – original draft (equal). **Donata Cafasso:** data curation (equal), formal analysis (equal), investigation (equal), methodology (equal), software (equal), validation (equal), writing – review and editing (equal). **Vito Antonio Romano:** conceptualization (equal), data curation (equal), investigation (equal), supervision (equal). **Katharina Brandt:** data curation (equal), formal analysis (equal), investigation (equal), methodology (equal), software (equal), validation (equal), writing – review and editing (equal). **Manfred Ayasse:** conceptualization (equal), data curation (equal), methodology (equal), supervision (equal), writing – review and editing (equal). **Salvatore Cozzolino:** conceptualization (equal), funding acquisition (lead), project administration (lead), supervision (equal), writing – original draft (equal), writing – review and editing (equal).

## Funding

M.S. was supported by a fellowship of the Italian National PhD Program on Artificial Intelligence, specialization area Agrifood and Environment, at the University of Naples Federico II. The research was conducted in the framework of the Project FRA‐HYSENSING granted to D.C. and was financially supported by the University of Naples Federico II.

## Conflicts of Interest

The authors declare no conflicts of interest.

## Supporting information


**Data S1:** ece374546‐sup‐0001‐DataS1.docx.

**Data S2:** ece374546‐sup‐0002‐DataS2.xlsx.

## Data Availability

The data that support the findings of this study are available in the Supporting Information of this article, and all raw data are also available as Data S1.
