# Supplementary material for: More Than Color: Floral Color Polymorphism Is Associated With Scent Differentiation and Pollination Success in the Deceptive Orchid Anacamptis pyramidalis
Source: Ecol Evol. 2026 Nov 2;16(11):e74546. doi: 10.1002/ece3.74546 (PMC13726758; doi:10.1002/ece3.74546)
Supplement: Supplementary file 1 [file ECE3-16-e74546-s001.docx]

**Supporting Information**

Table S1. Authentic reference standards used to confirm compound identities by GC–MS. Standards were analysed either individually or as custom mixtures under the same chromatographic conditions as floral samples.

|  | Authentic reference standards | |
| --- | --- | --- |
| Individual | | C7–C40 alkane standard mixture, C18, α-Pinene, Eucalyptol, Nonanal, Benzaldehyde, 1-Hexanol, (E)-β-Caryophyllene, Hexanal, Linalool, α-Terpineol, Acetophenone |
| Mix 1 | | 2-Ethyl-1-hexanol, Nonan-1-ol, Nerolidol, Tetradecan-1-ol, Hexadecyl acetate |
| Mix 2 | | Docosanal, Phenylacetaldehyde, Decanal, *p*-Anisaldehyde |
| Mix 3 | | (E)-Ocimene, Geranyl linalool, (+)-Sabinene, (Z,E)-α-Farnesene, Geraniol, α-Copaene |
| Mix 4 | | *p*-Pentadecanone, 6-Methyl-5-hepten-2-one, 4-Oxoisophorone |
| Mix 5 | | Methyl nicotinate, Methyl salicylate, Benzyl salicylate |

Table S2. Comparison between detected and literature RI of the main compounds.

| **Compound** | **detected RI** | **literature RI (HP-5MS/DB-5 equivalent)** |
| --- | --- | --- |
| Sabinene | 959.0 | ~969 |
| 6-Methyl-5-hepten-2-one | 973.3 | ~985 |
| Linalool | 1097.4 | ~1098 |
| 2-Phenylethyl alcohol | 1111.4 | ~1114 |
| Methyl nicotinate | 1132.1 | ~1138 |
| 4-Oxoisophorone | 1139.6 | ~1147 |
| Pentanal | ~700–710 | ~702 |
| (E)-Cinnamaldehyde | ~1265–1270 | ~1267 |
| Decanoic acid | 1346.8 | ~1367 |
| (E)-Cinnamyl acetate | 1377.3 | ~1385–1390 |
| Hexadecane | 1591.2 | ~1600 |
| 1-Nonadecene | 1685.9 | ~1890–1900* |
| Nonadecanol | 2273.2 | ~2260–2280 |
| Heptacosane | 2488.7 | ~2700 |
| Pentadecane | ~1500 | ~1500 |

B

A

Figure S1 Cytotype-related variation in floral VOC composition in Basilicata populations. (A) PCA of Hellinger-transformed relative VOC abundances and (B) boxplots of the six VOCs contributing most strongly to morph differentiation. Diploid and tetraploid individuals showed extensive overlap in multivariate space and no significant differences in the relative abundance of major compounds (all P > 0.05).

Figure S2. Mean relative abundance of selected morph-associated VOCs across geographically separated populations. Values represent mean relative abundances (%) calculated for each population-morph group. The displayed VOCs were selected from compounds contributing to floral colour morph differentiation in the four populations. Colours represent standardized abundances, expressed as z-scores calculated separately for each compound across groups.
